# Effectiveness of Computer-Tailored Health Communication in Increasing Physical Activity in People With or at Risk of Long-Term Conditions: Systematic Review and Meta-Analysis

**DOI:** 10.2196/46622

**Published:** 2023-10-04

**Authors:** Longdan Hao, Stefan Goetze, Tourkiah Alessa, Mark S Hawley

**Affiliations:** 1 Centre for Assistive Technology and Connected Healthcare School of Health and Related Research University of Sheffield Sheffield United Kingdom; 2 Department of Computer Science University of Sheffield Sheffield United Kingdom; 3 Biomedical Technology Department College of Applied Medical Sciences King Saud University Riyadh Saudi Arabia

**Keywords:** computer-tailored health communication, meta-analysis, long-term conditions, physical activity promotion

## Abstract

**Background:**

Regular physical activity (PA) is beneficial for enhancing and sustaining both physical and mental well-being as well as for the management of preexisting conditions. Computer-tailored health communication (CTHC) has been shown to be effective in increasing PA and many other health behavior changes in the general population. However, individuals with or at risk of long-term conditions face unique barriers that may limit the applicability of CTHC interventions to this population. Few studies have focused on this cohort, providing limited evidence for the effectiveness of CTHC in promoting PA.

**Objective:**

This systematic review and meta-analysis aims to assess the effectiveness of CTHC in increasing PA in individuals with or at risk of long-term conditions.

**Methods:**

A systematic review and meta-analysis were conducted to evaluate the effect of CTHC in increasing PA in people with or at risk of long-term conditions. Hedges *g* was used to calculate the mean effect size. The total effect size was pooled and weighted using inverse variance. When possible, potential moderator variables were synthesized, and their effectiveness was evaluated by subgroups analysis with *Q* test for between-group heterogeneity *Q_b_*. Potential moderator variables included *behavior change theories and models* providing the fundamental logic for CTHC design, *behavior change techniques and tailoring strategies* to compose messages, and *computer algorithms* to achieve tailoring. Several methods were used to examine potential publication bias in the results, including the funnel plot, Egger test, Begg test, fail-safe N test, and trim-and-fill method.

**Results:**

In total, 24 studies were included in the systematic review for qualitative analysis and 18 studies were included in the meta-analysis. Significant small to medium effect size values were found when comparing CTHC to general health information (Hedges *g*=0.16; *P*<.001) and to no information sent to participants (Hedges *g*=0.29; *P*<.001). Half of the included studies had a low to moderate risk of bias, and the remaining studies had a moderate to high risk of bias. Although the results of the meta-analysis indicated no evidence of publication bias, caution is required when drawing definitive conclusions due to the limited number of studies in each subgroup (N≤10). Message-tailoring strategies, implementation strategies, behavior change theories and models, and behavior change techniques were synthesized from the 24 studies. No strong evidence was found from subgroup analyses on the effectiveness of using particular behavior change theories and models or from using particular message-tailoring and implementation strategies.

**Conclusions:**

This study demonstrates that CTHC is effective in increasing PA for people with or at risk of long-term conditions, with significant small to medium effects compared with general health information or no information. Further studies are needed to guide design decisions for maximizing the effectiveness of CTHC.

## Introduction

### Long-Term Health Conditions and Physical Activity

A long-term health condition, also referred to as a chronic condition or chronic disease, “is a health problem that requires ongoing management over a period of years or decades” [1]. Unlike acute illnesses, long-term health conditions cannot be cured completely. However, they can be effectively controlled and managed through appropriate medical treatments, therapies, and lifestyle adjustments over the long term [1]. Long-term conditions caused more than 70% of global deaths in 2019. Seven out of the “top 10 causes of death” are long-term conditions: ischemic heart disease; stroke; chronic obstructive pulmonary disease; trachea, bronchus, and lung cancer; Alzheimer disease and other dementias; diabetes mellitus; and kidney disease [2].

Physical activity (PA) has been shown to improve symptoms in people with long-term conditions [3-7]. However, extra barriers caused by the symptoms of long-term conditions can reduce people’s adherence to the recommended PA level [6,8]. Tailored information and personalized feedback have been identified as effective facilitators for increasing PA [9].

### Computer-Tailored Health Communication

Computer technology has increasingly made tailoring feasible on a large scale, enabling access to a wide population [10-12]. Computer-tailored health communication (CTHC) uses computer-based platforms for individual information collection and processing, tailored information, and personalized feedback provision [10-12]. By embracing 3 different disciplines (computer science, health informatics, and behavior science) [10-12], CTHC has developed its own concepts: message-tailoring strategies and message implementation strategies [12]. *Message-tailoring strategies* cover two subcategories: (1) the tailoring criteria (based on different behavior change theories and models), such as the stage of change [13,14] or individual risk factors, and (2) tailoring mechanisms (what information is used and how it is used, such as demographic information for personalization and content adaptation, or performance records for feedback and training) [12]. *Message implementation strategies* include three subcategories: (1) general implementation strategies (how do messages depend on user assessment and tailoring iteration; will professionals be involved in message tailoring or technical support), (2) delivery modalities (eg, web technologies or traditional channels), and (3) tools for promoting behavior (eg, goal-setting tools, skill training, reminders, or monitoring tools) [12].

### The Effectiveness of CTHC in Promoting Health Behaviors

Previous systematic reviews have suggested that CTHC can provide promising results in behavior change interventions [15,16]. For the general population, CTHC has been shown to be more effective than general (nontailored) information on dietary promotion (eg, fruit and vegetable consumption and fat intake decrease) [17-21]; mammography screening [20,22]; smoking cessation [20]; and PA [17,20]. However, Lustria et al [15] suggested that CTHC has a larger impact on behavior change in healthy populations than in people with long-term conditions. Only 1 systematic review and meta-analysis has assessed tailored messages for people with a long-term condition, type 2 diabetes, and this review found CTHC to be more effective for self-management promotion than general health information for this condition [23].

These results indicate that CTHC may play a role in promoting greater PA in people with long-term conditions. However, the effectiveness of CTHC may vary across different populations, considering factors such as health status, age, and gender [15]. In addition, variations in effectiveness may be observed when considering different behaviors, such as dietary habits, smoking, PA, and screening tests for common diseases [15]. To the best of the authors’ knowledge, no reviews have been conducted to evaluate the effectiveness of CTHC systems on PA behavior change specifically for people with or at risk of long-term conditions; nor have reviews jointly analyzed psychological theories for behavior change and computer algorithms used in CTHC, and so there is limited information to understand how tailoring systems work and interact with individuals with long-term conditions as a whole. Given that increasing PA is a crucial aspect of self-management for individuals with long-term conditions [3], exploring the effectiveness of CTHC in this regard is valuable. Therefore, it is essential to comprehend not only the impact of CTHC on PA but also the influential factors contributing to its effectiveness.

The primary aim of this systematic review was to synthesize evidence on the effectiveness of CTHC on PA behavior change in people with or at risk of different long-term conditions. The secondary aims were to synthesize and evaluate message-tailoring strategies, message implementation strategies, and computer algorithms used in the included studies to provide suggestions to guide future CTHC designs for people with long-term conditions.

## Methods

### Inclusion and Exclusion Criteria

The Participants, Intervention, Comparison, Outcome, Study framework [24] was used to structure the inclusion and exclusion criteria (for details, see Textbox 1).

**Inclusion and exclusion criteria.**

**Inclusion criteria**

1. Participants

1.1. Adult population (aged ≥18 years).

1.2. People with long-term conditions (in total, 103 long-term conditions [25] were identified for study selection) or people with a BMI of ≥25 kg/m^2^ (overweight). This review includes people with overweight and obesity since the risk of having long-term conditions increases with a BMI of ≥25 kg/m^2^ [26-28].

2. Intervention

2.1. Participants receive tailored messages to promote physical activity (PA). Tailored messages are defined as individualized and customized to fit the participants’ personal needs, status, interests, and concerns. Tailored messages follow at least one type of tailoring strategy, such as personalization (eg, mentioning the participant’s name) or feedback on PA performance. Messages are tailored according to (1) the participant’s personal characteristics, such as demographics, preferences, and physical and psychological status, or (2) behavior performance, preferences, and barriers for PA. Tailored messages can be combined with standard PA interventions including rehabilitation programs, self-management programs, and eHealth interventions. Message channels include SMS text messages, email, websites, smartphone apps, printed materials, fliers and other channels.

2.2. Tailored messages are generated or selected by computer algorithms.

3. Comparison

3.1. Participants in the control group receive general information (nontailored messages) or receive no messages.

4. Outcome

4.1. The outcome must include at least one measure reflecting PA change. PA could be measured as activity performance (eg, steps), activity length and frequency (eg, minutes, times/week), or energy consumption.

5. Study

5.1. Only randomized controlled trials (RCTs) are included.

5.2. Journal papers

5.3. Written in English

**Exclusion criteria**

1. Participants

1.1. Children (aged <18 years, unless national law defines a person as being an adult at an earlier age).

1.2. Healthy population with a BMI <25 kg/m^2^.

2. Intervention

2.1. Tailored messages are targeted to promote behaviors other than PA. Messages are population based or community based (unlike individualized messages, population-based or community-based messages provide the same content to the whole targeted population or a community);

2.2. Tailored messages are generated or selected by humans, for example, health professionals.

3. Comparison

3.1. Absence of a control group

4. Outcome

4.1. Only qualitative outcomes or indirect outcomes of PA

5. Study

5.1. Non-RCTs (eg, reviews, pilot studies, feasibility studies, and single-arm studies)

5.2. Protocols, reviews, unpublished studies, conference abstracts, posters, theses, and articles

5.3. Written in languages other than English

### Search Method

We used search keywords in initial searches in Embase (OVID). After developing the search strategy (for details, see Multimedia Appendix 1), an adaptation was made for other databases. Studies from the earliest date in the selected databases until the search date (February 18, 2023) were included.

Four keywords were used to develop Medical Subject Headings terms in the search strategy: (1) PA and exercise, (2) chronic disease, (3) tailored message, and (4) randomized controlled trial in OVID. The electronic databases included MEDLINE (OVID), Embase (OVID), PsycINFO (OVID), CINAHL, the Cochrane Central Register of Controlled Trials (The Cochrane Library), and the Web of Science Core Collection.

### Study Selection, Data Extraction, and Management

After duplicate studies were removed, three steps were followed for selecting and analyzing the remaining studies: (1) title screening, (2) abstract screening, and (3) full-text screening. At the beginning of title screening, 2 assessors (LH and TA) independently screened the first 20 studies ordered alphabetically and discussed the results for a deeper understanding of the inclusion and exclusion criteria. For all studies, each assessor graded the studies independently: 0 for exclusion, 1 for not sure, and 2 for inclusion. Studies were included in the next screening if the sum of the grades was ≥2. Independent screening of the title, abstract, and full content was conducted successively. After each screening, the assessors discussed the difference in the results. If any disagreement persisted after the discussion, the final decision was made by a third assessor (MH).

The main assessor (LH) extracted study characteristics, intervention details, and outcomes. The risk of bias was assessed by using the Cochrane risk of bias tools [29] (*Risk of bias* section).

Study characteristics included the number of randomized controlled trial (RCT) arms (study design), duration, country of intervention, diagnosis of the participants, age, gender, and sample size. Intervention details included information channel, baseline assessment for message tailoring, message delivery frequency and time (not applicable for post and website log-in), message type (text, video, hyperlink, and external resources), and direction (1-way messages and 2-way messages), behavior change theories applied in the study, behavior change techniques (BCTs; based on 93 BCTs taxonomy defined by Michie et al [30]), tailoring on aspects of individuals, computer algorithms for achieving message generation and selection, and professional involvement. Outcomes included the types of PA, follow-up assessment time, and measurement tools.

### Data Collection and Analysis

#### Meta-Analytic Approach

The goal of the meta-analysis was to examine the effect of tailored messages on the level of PA. Studies were categorized based on the types of comparison groups: a nontailored control group (a control group receiving general health messages) and a nontreatment control group (a control group receiving no messages).

The calculations were supported by Review Manager 5.4 (RevMan, provided by Cochrane). The meta-analysis approach used standardized mean difference with a random effects model and inverse-variance weighted and pooled effect size (standard mean difference between 2 comparison groups) [31] across studies. If the studies reported multiple assessment points in time for PA outcomes, the final point in time was used. This procedure is consistent with that used in other studies [20]. If studies reported multiple assessment methods for PA, objective continuous outcomes (eg, daily steps measured by pedometers) reported by most studies were chosen first to minimize heterogeneity.

Eligible studies were analyzed using the mean difference and SD to measure the change from baseline to the last end point in the intervention period. The effect size of continuous PA outcomes with different measures was calculated together with Hedges adjusted *g*. This step increases the heterogeneity of the result. However, owing to the limited number of tailoring studies and nonstandard methodologies, this calculation has been widely applied in previous meta-analyses on tailoring intervention studies. The effect size was interpreted as small (Hedges *g*=0.2), medium (Hedges *g*=0.5), or large (Hedges *g*=0.8) [32].

Heterogeneity in this study is presented by *I*^2^ [29]. Compared with the chi-square test for heterogeneity, *I*^2^ measures the impact of heterogeneity on the meta-analysis (the percentage of the variability in effect size that is caused by heterogeneity) [29]. Heterogeneity *I*^2^ is classified as low (0%≤*I*^2^<25%), medium (25%≤*I*^2^<75%), or high (*I*^2^≥75%) [33].

#### Between-Groups Analysis Approach

Between-groups analysis considered the main behavioral change theories and models reported in tailoring designs of included studies, the number of reported behavioral change theories, and types of outcome measurement (objective or subjective). The potential moderators for between-groups analysis were first translated into dichotomous values (presence or absence), and the average effect size was calculated when a minimum of 3 studies could be included at both levels of the moderators. A Q test for between-groups heterogeneity (*Q_b_*) was used to measure the significance of the difference between subgroups. *Q_b_* was calculated by the chi-square test with *df*=*n–1* (n being the number of subgroups) [34,35].

#### Qualitative Analysis Approach

The qualitative analysis aimed to understand the design and implementation of tailoring interventions and the effective elements of behavior change interventions. The qualitative approach focuses on three subcategories: (1) message-tailoring strategies (behavior change theories and models and BCTs); (2) message implementation strategies (delivery channel, iteration, message type, direction, format, frequency, timing, and content); and (3) computer algorithms. Applied strategies were summarized, cumulated, and categorized if possible.

### Risk of Bias

Risk of bias was assessed based on Cochrane Collaboration’s Risk of Bias Tools [29]. These tools are used to assess the study biases caused by the study design and execution and their impact on the findings of the study [29]. For each study, the main reviewer assessed the risk of bias using the Cochrane Collaboration’s Risk of Bias tool and criteria. The criteria evaluated various aspects of the included studies, such asselection bias, performance bias, detection bias, attrition bias, and reporting bias. Each criterion was assessed as having a low, high, or unclear risk of bias based on the information provided in the study report or other sources. For example, the selection bias criterion for a RCT would assess the method of random sequence generation, allocation concealment, and baseline comparability of the groups. The overall risk of bias for each study was then classified as low, high, or unclear based on the judgments for each criterion. Studies with a low risk of bias were considered to have a low likelihood of bias affecting the study results, whereas studies with a high risk of bias were considered to have a high likelihood of bias affecting the study results. Studies with an unclear risk of bias had insufficient information to make a definitive judgment.

### Publication Bias

Given the small sample size and high heterogeneity of the included studies, we assessed publication bias using a range of methods, including funnel plot testing, Egger test, Begg test, the trim-and-fill method, and the fail-safe N test, as appropriate, given the known limitations of these methods. These analyses were conducted using the meta package in Stata/MP (version 17; StataCorp) [29,36].

## Results

### Description of the Studies

#### Overview

**Figure 1:**
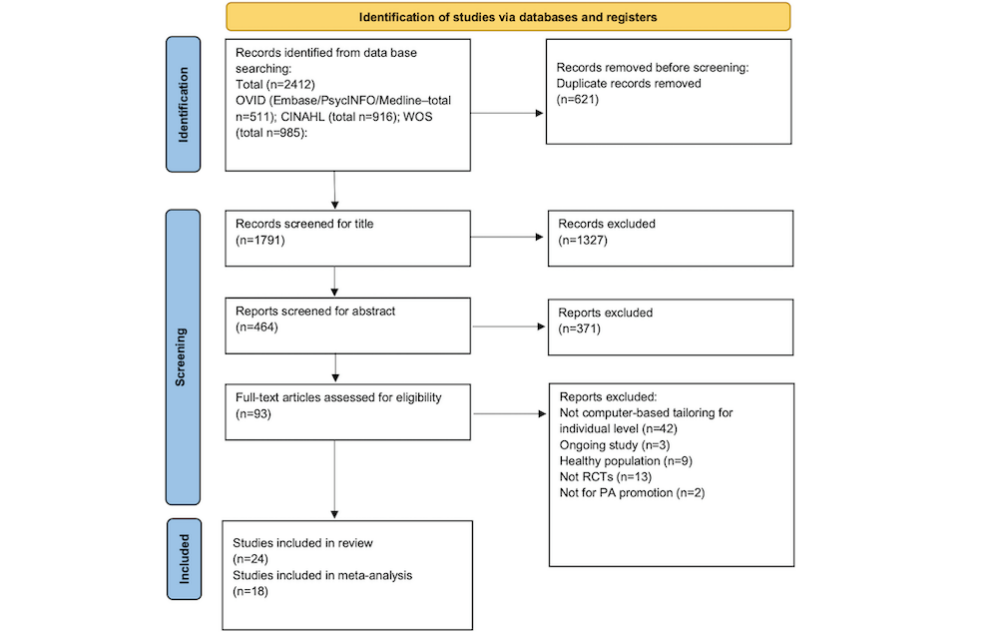
Updated version of PRISMA (Preferred Reporting Items for Systematic Reviews and Meta-Analyses) flow diagram of the literature search and selection process—computer-tailored health communication intervention on increasing physical activity (PA) in people with or at risk of long-term conditions. RCT: randomized controlled trial.

A total of 1791 studies were identified from 5 electronic databases after duplicates (n=620) were removed. Figure 1 shows the selection process based on the PRISMA (Preferred Reporting Items for Systematic Reviews and Meta-Analyses) guidelines [29,37]. Following title screening, 464 studies were retained for abstract screening and 93 studies were subsequently taken forward for screening of the complete article. In total, 24 studies were identified to meet the eligibility criteria for inclusion in this review. Agreement between the assessors was very high (Cohen κ=0.811). Among the included studies, 2 articles [38,39] reported results based on the same RCT but with different assessment times. Five articles [38,40-43] reported the details of the study design in separate papers. Therefore, the data reported in these separate papers were extracted if necessary. The details of PRISMA checklist is reported in Multimedia Appendix 2.

#### Study Characteristics

All 24 studies [17,39,41-61] included in this review were RCTs published in English between 1999 and 2023. Details of the selected studies are summarized in Table 1. In total, 19 studies were 2-arm RCTs, 3 studies were 3-arm RCTs, and 2 studies were 4-arm RCTs. The sample sizes ranged from 36 to 1366, resulting in a total sample size of 11,754. Studies had an average duration of 10 months. The study population was aged 55.7 (SD 8.67) years on average, and 57.6% (6771/11,754) were female. All participants in the included studies had or were at risk of long-term conditions, including cardiovascular diseases (8 studies), cancer and survivors of cancer (7 studies), diabetes (8 studies), chronic obstructive pulmonary disease (1 study), and overweight or obesity (1 study).

**Table 1:** Details of the 24 selected studies.

Study and year	Sample size, n	Country of setting	Length (months)	RCT^a^ arms	Diagnosis of participants	Age (years), mean (SD)	Gender (female), %
Agboola et al [44], 2016	126	United States	6	2	Type 2 diabetes	51.5 (11.6)	51.6
Almeida et al [45], 2015	452	United States	1 out of 6^b^	4	Sedentary cardiac patients	58.6 (9.7)	59.1
Antypas and Wangberg [46], 2014	69	Norway	22	2	Cardiac diseases	59.1 (8.7)	21.7
Beleigoli et al [47], 2020	1298	Brazil	6	3	BMI ≥25 kg/m^2^	33.6 (11.0)	76.7
Block et al [48], 2016	339	United States	6	2	Prediabetes	55 (8.9)	31.0
Boudreau et al [49], 2011	325	Canada	8	2	Type 2 diabetes	49.1 (6.6)	44.3
Broekhuizen et al [17], 2012	340	The Netherlands	12	2	Familial hypercholesterolemia	45.9 (12.9)	56.8
Bull et al [50], 1999	272	United States	3	4	Overweight sedentary patients with risk of cardiovascular diseases	39.6 (13.3)	63.2
Demark-Wahnefried et al [38] and Demark-Wahnefried [51], 2007	543	United States	10	2	Survivors of breast and prostate cancer	57 (10.8)	56.0
Dobson et al [40], 2018	366	New Zealand	9	2	Poorly controlled diabetes	47 (15.0)	48.4
Frederix et al [52], 2015	140	Belgium	6	2	Cardiac patients	61 (8.5)	18.6
Golsteijn et al [41], 2018	478	The Netherlands	4	2	Patients with and survivors of prostate and colorectal cancer	66.5 (7.6)	13.0
Gomersall et al [53], 2019	36	Australia	3	2	People with cancer and survivors	64.8 (9.6)	36.1
Kanera et al [42], 2017	462	The Netherlands	12	2	Survivors of cancer	55.9 (11.4)	79.9
Khunti et al [54], 2021	1366	England	48	3	People at risk of diabetes	61 (9.1)	48.8
Migneault et al [55], 2012	337	United States	8	2	African American individuals with hypertension	56.6 (11.0)	70.3
Ottenbacher et al [39], 2012^c^	400	United States	12	2	Survivors of breast and prostate cancer	57 (11.1)	56.0
Peacock et al [62], 2020	204	United Kingdom	6	2	People at risk of cardiovascular disease and type 2 diabetes	64 (6.0)	35.8
Rock et al [43], 2015	693	United States	24 out of 48	2	Any early-stage breast cancer	56.3 (9)	100.0
Short et al [57], 2015	330	Australia	3	3	Survivors of breast cancer	55 (—)^d^	100.0
Storm et al [58], 2016	790	Germany and the Netherlands	8	2	Cardiac patients	50.8 (12.2)	62.9
Vluggen et al [59], 2021	478	The Netherlands	6	2	Patients with T2DM^e^	60 (6.76)	32.4
Volders et al [60], 2020	585	The Netherlands	12	2	Older adults with chronic illnesses	74.5 (6.4)	48.4
Voncken-Brewster et al [61], 2015	1325	The Netherlands	6	2	People at risk for or diagnosed with COPD^f^	57.6 (7.2)	52.7

^a^RCT: randomized controlled trial.

^b^The length of study was 6 months in total. However the reported data collected from follow-up assessment was at the end of the first month.

^c^2-year follow-up study of Demark-Wahnefried [47].

^d^SD is not available in the paper.

^e^T2DM: type 2 diabetes mellitus.

^f^COPD: chronic obstructive pulmonary disease.

### Risk of Bias

**Figure 2:**
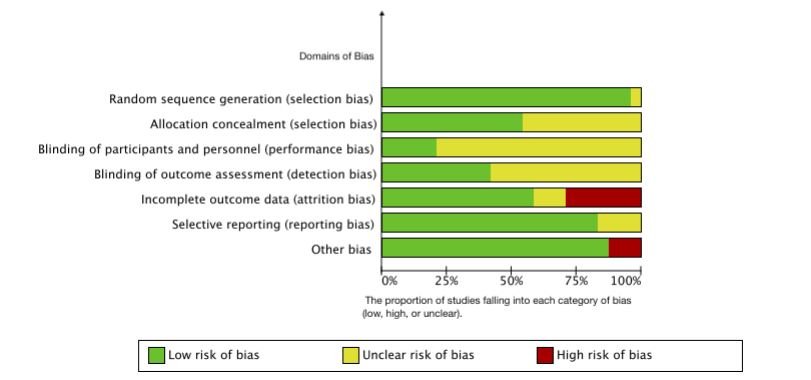
Summary of risk of bias.

**Figure 3:**
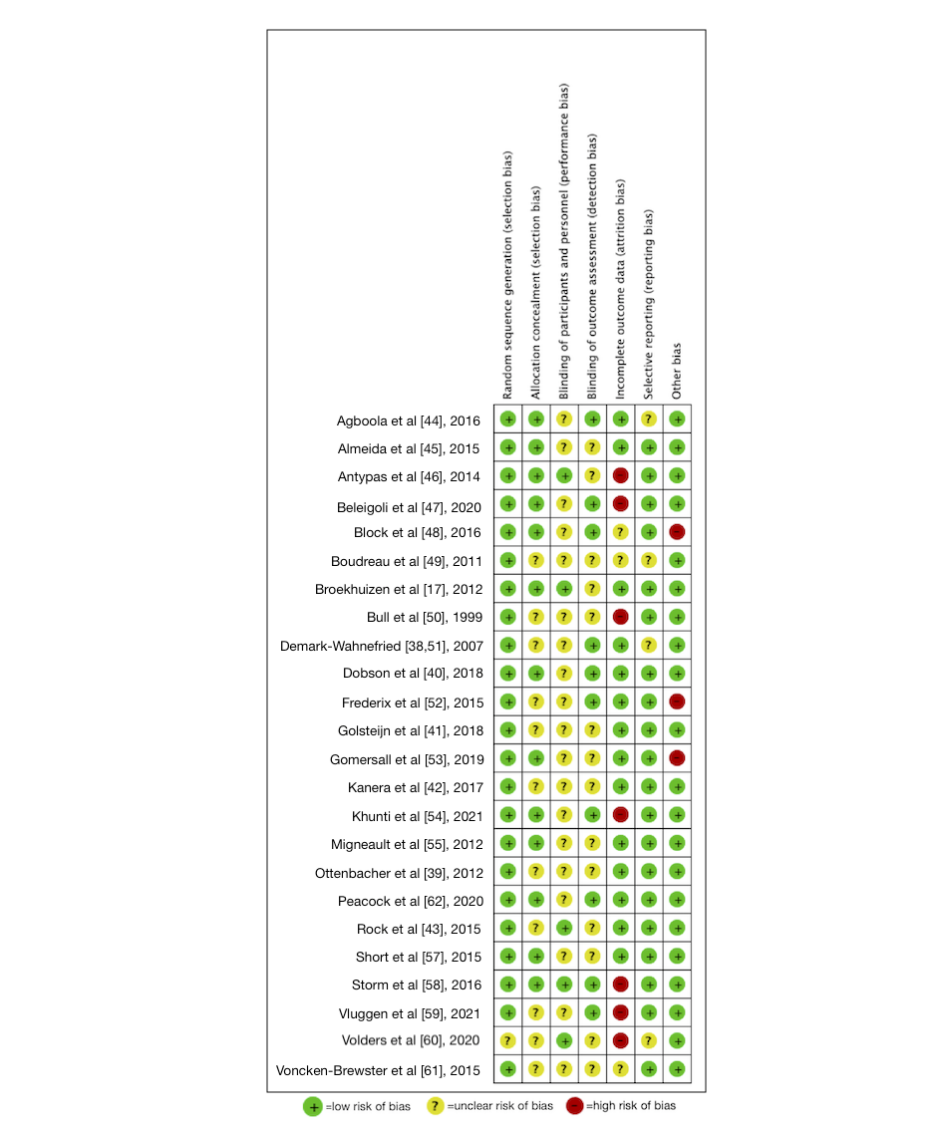
Risk of bias assessments for studies in a systematic review of computer-tailored health communication on increasing physical activity for people with chronic obstructive pulmonary disease. Key: +=low risk of bias; ?=unclear risk of bias; −=high risk of bias.

All included studies presented some degree of potential bias when assessed using the Cochrane Collaboration Risk of Bias Tool. Figure 2 shows the degree of risk of bias, and Figure 3 [17,38-55,57-62] shows the identified risks in each study.

In total, 12 studies were considered to have a moderate to high risk of bias [46-48,50,52-54,58-61,63], and the remaining 12 studies were considered to have a low to moderate risk of bias. All the studies reported random sequence generation, except for Volders et al [60], which did not report details on randomization. In total, 13 studies concealed the allocation until the trials started. Five studies [17,43,46,58,60] blinded the participants, and 10 studies [38,40,44,47,48,52,54,56,58,59] blinded the outcome assessors. Fourteen studies had a low attrition rate or found no difference in intention-to-treat assessment, while 7 studies had a very high attrition rate [46,47,50,54,58-60]. In total, 21 studies reported the study registration and study design. Two studies have a high risk of bias in other areas owing to the small sample size.

### Meta-Analysis on PA Outcomes

After the study characteristics were summarized from 24 studies, 6 studies were excluded from the meta-analysis for the following reasons: Bull et al [50] and Agboola et al [44] provided different types of outcomes (dichotomous outcomes and log of risk ratio) to most studies. It was not possible to combine these 3 outcomes with other outcomes. Limited information was provided by Boudreau et al [49], Migneault et al [55], and Voncken-Brewster et al [61] to calculate the standardized mean difference between the groups. The effect size reported by Broekhuizen et al [17] was much larger than that reported in other studies and the SE was very small. This resulted in a significant increase in overall heterogeneity. Therefore, the study by Broekhuizen et al [17] was considered as an outlier and was excluded from the meta-analysis.

**Figure 4:**
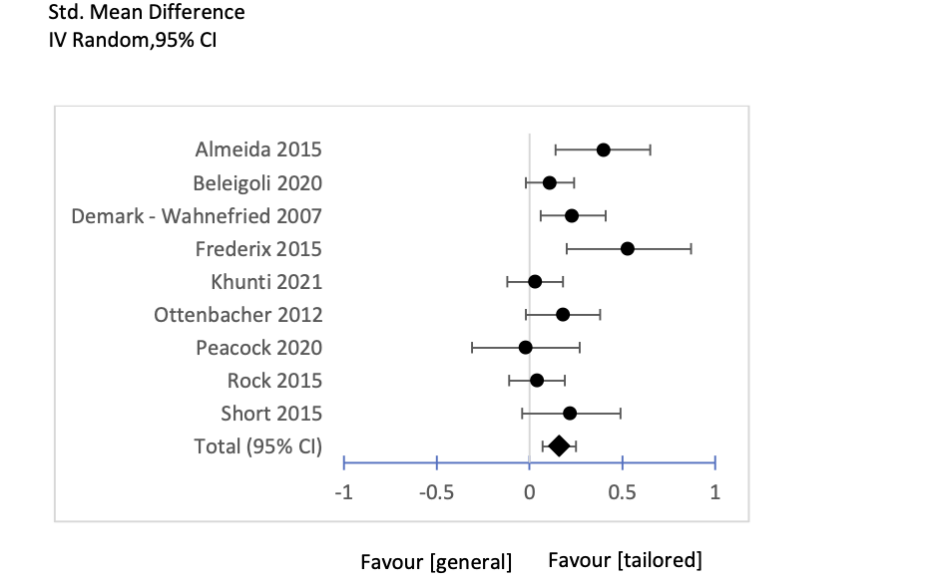
Subgroup 3 Forest Plot tailored message or General message.

**Figure 5:**
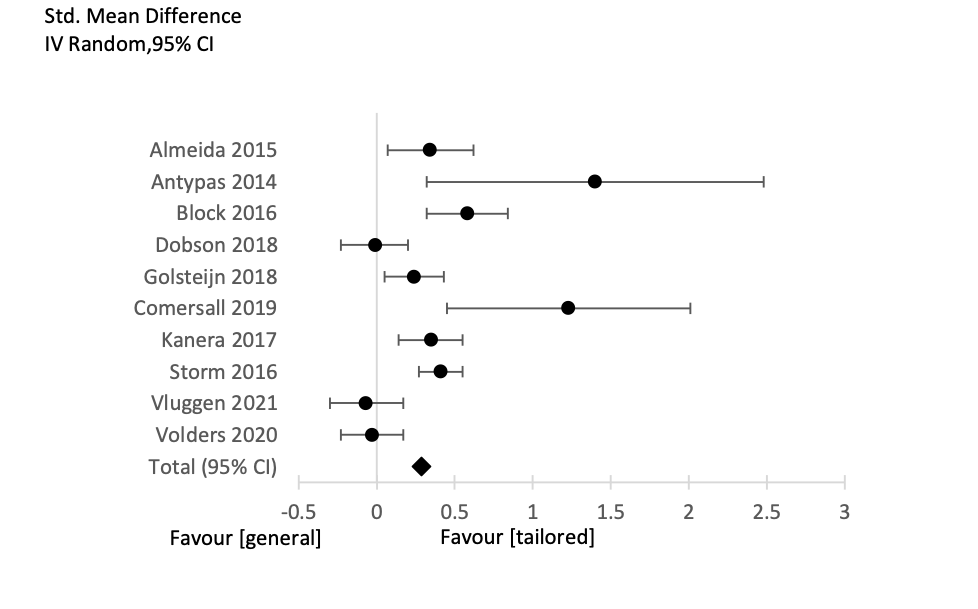
Subgroup 3 Forest Plot tailored message or No message.

A total of 18 studies were included in the meta-analysis and were categorized into two subgroups to be analyzed using forest plots as follows:

1. Subgroup 1: intervention group with tailored messages versus control group with general messages (tailored vs general): 9 studies were included in this subgroup; the results and forest plot are shown in Table 2 and Figure 4.
2. Subgroup 2: intervention group with tailored messages versus control group with no message (tailored vs none): 10 studies were included in this subgroup; the results and forest plot are shown in Table 3 and Figure 5.

**Table 2:** Subgroup 1: forest plot of tailored versus general messages^a^.

Study of subgroup, year	Experimental		Control			Standard mean difference		
	Values, mean (SD)	Total^b^, n	Values, mean (SD)	Total, n	Weight (%)		IV^c^	Random, 95% CI
Almeida et al [45], 2015	109.71 (89.33)	125	74.04 (89.76)	115	8.5		0.40	0.14 to 0.65
Beleigoli et al [47], 2020	2.7 (3.13)	420	2.4 (2.21)	470	16.2		0.11	−0.02 to 0.24
Demark-Wahnefried et al [38] Demark-Wahnefried [51], 2007	112.7 (126.6)	253	83.8 (119.1)	266	13.1		0.23	0.06 to 0.41
Frederix et al [52], 2015	2077 (2357)	69	1075 (1203)	70	5.7		0.53	0.20 to 0.87
Khunti et al [54], 2021	−296 (2969)	317	−385 (2217)	373	14.8		0.03	−0.12 to 0.18
Ottenbacher et al [39], 2012	60 (180)	171	30 (150)	229	11.5		0.18	−0.02 to 0.38
Peacock et al [62], 2020	161.9 (58.12)	134	163 (58.12)	70	7.2		−0.02	−0.31 to 0.27
Rock et al [43], 2015	165 (185.2)	343	157 (205.2)	348	14.9		0.04	−0.11 to 0.19
Short et al [57], 2015	9644.8 (7745.6)	109	8301.2 (3373.4)	110	8.1		0.22	−0.04 to 0.49
Total	N/A^d^	1941	N/A	2051	100		0.16^e^	0.07 to 0.25

^a^Heterogeneity: τ^2^=0.01 (an estimate of the between-study variance in a random effects meta-analysis [τ^2^]. The square root of this number [ie, τ] is the estimated SD of the underlying effects across studies); *χ*^2^_8_=15.8; P=.05; *I*^2^=49%. Test for overall effect (*Z* test and P value of standardized mean difference): *Z*=3.43 (*P*<.001).

^b^Number of participants in the treatment and control group of each study.

^c^IV: inverse variance.

^d^N/A: not applicable.

^e^Overall effect.

**Table 3:** Subgroup 2: forest plot of tailored versus no messages^a^.

Study of subgroup, year	Experimental			Control			Standard mean difference		
	Values, mean (SD)	Total^b^, n	Values, mean (SD)		Total, n	Weight (%)		IV^c^	Random effect, 95% CI
Almeida et al [45], 2015	108.46 (89.046)	105	77.97 (89.0058)		103	10.6		0.34	0.07 to 0.62
Antypas and Wangberg [46], 2014	5613 (2828)	7	1356 (2937)		11	2.2		1.4	0.32 to 2.48
Block et al [48], 2016	1.21 (1.3607)	100	0.42 (1.3496)		147	10.9		0.58	0.32 to 0.84
Dobson et al [40], 2018	3.45 (2.03)	169	3.48 (2.19)		172	11.8		−0.01	−0.23 to 0.2
Golsteijn et al [41], 2018	1145 (883)	222	943 (769)		213	12.3		0.24	0.05 to 0.43
Gomersall et al [53], 2019	17.1 (54.0477)	16	−50.1 (52.3672)		15	3.7		1.23	0.45 to 2.01
Kanera et al [42], 2017	688.1 (570.6)	162	512.2 (452.1)		206	11.9		0.35	0.14 to 0.55
Storm et al [58], 2016	1 (1.66)	403	0.34 (1.53)		387	13.2		0.41	0.27 to 0.55
Vluggen et al [59], 2021	833 (741)	111	884 (777)		177	11.3		−0.07	−0.30 to 0.17
Volders et al [60], 2020	200 (206)	164	206 (197)		246	12.1		−0.03	−0.23 to 0.17
Total	N/A^d^	1459	N/A		1677	100		0.29^e^	0.12 to 0.46

^a^Heterogeneity: τ^2^=0.05 (an estimate of the between-study variance in a random effects meta-analysis [τ^2^]. The square root of this number [ie, τ] is the estimated SD of the underlying effects across studies); *χ*^2^_9_=43.6; *P*<.001; *I*^2^=79%. Test for overall effect (*Z* test and P value of standardized mean difference): *Z*=3.43 (*P*<.001).

^b^Number of participants in treatment and control group of each study.

^c^IV: inverse variance.

^d^N/A: not applicable.

^e^Overall effect.

Subgroup 1 (tailored vs general messages; Table 2) showed a small statistically significant weighted mean effect size between the treatment and control groups (Hedges *g*=0.16, *P*<.001, 95% CI 0.07-0.25). In total, 9 studies with 1941 participants in the intervention groups and 2051 participants in the control groups were included. The heterogeneity [64] among these studies was medium (*I*^2^=49%).

In subgroup 2 (tailored vs no messages; Table 3), a small to medium and statistically significant weighted mean effect size was observed between the treatment and control groups (Hedges *g*=0.29, P=.001, 95% CI 0.12-0.46). In total, 10 studies with 1459 participants in the intervention groups (“Total” in column 4) and 1677 participants in the control groups were included. The heterogeneity [64] in this subgroup was high (*I*^2^=79%).

In both between-groups analyses, heterogeneity [29] was caused by the diversity of participants, different types of long-term conditions, the different tailoring strategies and implementation strategies, and the different methods of PA measurement.

### Publication Bias

The funnel plot analysis for both meta-analyses (tailored vs general messages and tailored vs no messages interventions) did not reveal any significant asymmetry. The effect size estimate obtained from the fill-and-trim method (observed+imputed: effect size=0.139, 95% CI 0.036-0.242 for tailored vs no message interventions; effect size=0.225, 95% CI 0.003-0.447 for tailored vs general messages) did not differ significantly from the effect size estimate obtained from the meta-analysis, and the Begg test supported the null hypothesis H0 of no small study effect for both analyses (P=.59). In contrast, Egger test rejected the none hypothesis of no small study effect for both meta-analysis: for tailored versus no messages (P=.05) and for tailored versus general messages (P=.02). It is important to note that the power of all methods used to test for the risk of publication bias was low because of the limited number of studies (n=10); thus, caution is needed when drawing conclusions from the results [36] (Multimedia Appendix 3: output data for publication bias analysis).

### Message-Tailoring Strategies

The message-tailoring strategies applied in the 24 studies were analyzed to provide suggestions for designing future tailoring studies. *Behavior change theories* and *behavior change techniques* were extracted from the 24 included studies.

#### Behavior Change Theories and Models

In total, 22 behavior change theories and models were identified in the studies. The most frequently reported theories were social cognitive theory (SCT; 9 studies) [13], the I-change model (6 studies) [65], the stage of change in the transtheoretical model (5 studies) [13,14], the health action process approach (HAPA; 4 studies) [66], and the theory of planned behavior (3 studies) [13,67]. The impact of each behavior change theory or model mentioned earlier on effect sizes was tested using *Q_b_* (Table 4). In subgroup 1, the effect size for studies using SCT was higher than that of studies without SCT. However, the opposite result was obtained in subgroup 2, and neither difference reached significance (*Q_b_*=0.42, P=.52 in subgroup 1 and *Q_b_*=0.08, P=.77 in subgroup 2). Few studies used the I-change model or HAPA in subgroup 1 for a between-groups analysis. In subgroup 2, the effect size for studies without the I-change model was higher than that for studies with the I-change model, and the difference was significant (*Q_b_*=3.75; P=.05). The effect size for studies using HAPA in subgroup 2 was higher than that of studies without HAPA. However, no difference reached significance (*Q_b_*=0.66; P=.42). Few studies have used the stage of change model and the theory of planned behavior for a between-groups analysis.

Comparing studies with different numbers of behavior change theories or models (none or one vs multiple), no differences reached significance in between-groups analyses (Table 5).

**Table 4:** Between-groups analysis on behavior change theories and models.

Tailoring strategy		Subgroup 1: tailored or general^a^					Subgroup 2: tailored or none^a^			
		Studies, n	Sample size, n	Standard mean difference (95% CI)	P value	Studies, n		Sample size, n	Standard mean difference (95% CI)	P value
**Test for subgroup differences between interventions with and without applying SCT^b^**										
	SCT	5	2069	0.19 (0.08 to 0.31)	.001	5		1599	0.29 (0.11 to 0.47)	.002
	No SCT	4	1923	0.13 (−0.04 to 0.29)	.13	5		1537	0.35 (−0.01 to 0.70)	.05
**Test for subgroup differences between interventions with and without applying I-CHANGE**										
	I-CHANGE	N/A^c^	N/A	N/A	N/A	4		1501	0.13 (−0.07 to 0.32)	.20
	No I-CHANGE	N/A	N/A	N/A	N/A	6		1635	0.45 (0.19 to 0.71)	<.001
**Test for subgroup differences between interventions with and without applying HAPA^d^**										
	HAPA	N/A	N/A	N/A	N/A	3		1243	0.38 (0.14 to 0.62)	.002
	No HAPA	N/A	N/A	N/A	N/A	7		1893	0.25 (0.02 to 0.47)	.03

^a^Test for subgroup differences between interventions with and without applying social cognitive theory: *χ*^2^_1_=0.4, P=.52, *I*^2^=0% for subgroup 1; *χ*^2^_1_=0.1, P=.77, *I^2^*=0% for subgroup 2. Test for subgroup differences between interventions with and without applying I-CHANGE: *χ*^2^_1_=3.8, P=.05, *I*^2^=73.4% for subgroup 2. Test for subgroup differences between interventions with and without applying health action process approach: *χ*^2^_1_=0.66, P=.42, *I*^2^=0% for subgroup 2.

^b^SCT: social cognitive theory.

^c^N/A: not applicable.

^d^HAPA: health action process approach.

**Table 5:** Between-groups analysis—using multiple behavior change theories to single and none.

		Subgroup 1: tailored or general^a^				Subgroup 2: tailored or none^a^			
		Studies, n	Sample size, n	Standardized mean difference (95% CI)	P value	Studies, n	Sample size, n	Standardized mean difference (95% CI)	P value
**Test for subgroup differences**									
	Multiple	3	1159	0.25 (0.13 to 0.37)	<.001	7	2027	0.27 (0.08 to 0.46)	.006
	Single or none	6	2833	0.11 (0.00 to 0.22)	.04	3	1109	0.39 (−0.08 to 0.86)	.11

^a^Test for subgroup differences: *χ*^2^_1_=2.9, P=.09, *I*^2^=65.7% for subgroup 1; *χ*^2^_1_=0.2, P=.64, *I*^2^=0% for subgroup 2.

#### Behavior Change Techniques

Thirty-one BCTs were identified based on the 93 BCTs taxonomy [30]. The number of identified BCTs in studies was between 3 and 12 BCTs. Most studies conducted before 2020 did not clarify the BCTs used in tailoring design; therefore, a high probability remains that the list of identified BCTs is incomplete. Hence, the applied BCTs are presented as a list in Textbox 2, instead of the accumulated numbers of use.

**Summary of behavior change techniques applied in selected studies based on 93 hierarchically clustered techniques (note that the numbering follows that from the study by Michie et al [30]).**

**1. Goal setting and planning**

1.1. Goal setting (behavior)

1.2. Problem solving

1.4. Action planning

1.5. Review behavior goals

1.6. Discrepancy between current behavior and goal

1.7. Review outcome goals

**2. Feedback and monitoring**

2.2. Feedback on behavior

2.3. Self-monitoring of behavior

2.4. Self-monitoring of outcomes of behavior

**3. Social support**

3.1. Social support (unspecified)

3.2. Social support (practical)

3.3. Social support (emotional)

**4. Shaping knowledge**

4.1. Instruction on how to perform a behavior

4.2. Information about antecedents

**5. Natural consequences**

5.1. Information about health consequences

5.3. Information about social and environmental consequences

5.6. Information about emotional consequences

**6. Comparison of behavior**

6.1. Demonstration of the behavior

6.2. Social comparison

**7. Associations**

7.1. Prompts or cues

**9. Comparison of outcomes**

9.2. Pros and cons

9.3. Comparative imagining of future outcomes

**10. Reward and threat**

10.1. Material incentive (behavior)

**11. Regulation**

11.2. Reduce negative emotions

**12. Antecedents**

12.1. Restructuring the physical environment

12.2. Restructuring the social environment

12.3. Avoidance or reducing exposure to cues for the behavior

**13. Identity**

13.2. Framing or reframing

**15. Self-belief**

15.1. Verbal persuasion about capability

15.3. Building self-belief

**16. Antecedents**

16.3. Imaginary reward

#### Tailoring on Different Characteristics of Individuals

On the basis of different behavior change theories and models, studies chose to tailor messages on different characteristics of individuals. This study synthesized the individuals’ characteristics used in tailoring to provide suggestions for future CTHC studies.

In total, 6 different categories were identified from the included studies (Textbox 3): barriers, personal characteristics, planning, disease, psychological factors, and performance. Tailoring on barriers to PA (1.1), personal goals (3.4), motives for increasing PA (5.6), stage of behavior change (5.12), and performance regarding the goal (6.7) were seen in most of the included studies.

**Categorizing tailoring information on different aspects of individuals.**

**Category, subgroup first layer, and subgroup second layer**

1. Barriers

1.1. Barriers for physical activity (PA)

2. Personal characteristics

2.1. Demographic information

2.1.1 Ethnic mapping

2.2. Cultural characteristics

2.2.1. African American voice

2.2.2. Tone and language

2.2.3. Content and wording

2.3. Personal determinant

2.4. Signed off using the first name of the coach

2.5. Select the timing, duration and frequency of messages and reminders

2.6. Weight

3. Planning

3.1. Action planning

3.2. Coping planning

3.3. Goal setting

3.4. Personal goals

4. Disease

4.1. Cancer-coping style

4.2. Comorbid conditions

5. Psychological factors

5.1. Risk

5.1.1. Awareness of the disease risk

5.1.2. Change in risk perception

5.1.3. Risk factor profiling

5.2. Cues to action

5.3. Exercise preferences

5.4. Motivational modifications

5.5. Moral obligation

5.6. Motives for increasing PA

5.7. Motivational determinants or constructs

5.7.1. Attitude

5.7.2. Intrinsic motivation

5.7.3. Self-efficacy

5.7.4. Social influences

5.7.5. Intention

5.8. Self-regulation

5.9. Pros and cons of being more physically active.

5.10. Response efficacy

5.11. Social support

5.12. Stage of behavior change

5.13. Outcome expectations

5.14. User’s values

6. Performance

6.1. Awareness of one’s own performance

6.2. Ability to prepare and execute plans to achieve goals and to overcome potential barriers

6.3. Baseline behavioral information

6.4. Behavioral modifications

6.5. Daily performance steps

6.6. PA level

6.7. Performance regarding the goal

### Message Implementation Strategies

This review summarizes two categories of applied message implementation strategies: (1) assessment on individuals and message iteration and (2) message delivery channel, direction, and frequency.

#### Assessment and Iteration

All the studies conducted baseline assessments for message tailoring. Baseline assessments included sociodemographic information, lifestyle (PA level, smoking status, and diet), psychological characteristics (stage of change, anxiety, PA motives, and attitude toward PA), health status (BMI and long-term conditions), and user habits regarding the use of websites. Ten studies [41,43,44,52-55,57,60,62] used *objective* assessment of PA (pedometer or accelerometry) and 14 studies [17,39,40,42,45-51,58,59,61] collected PA data through *subjective* questionnaires. In 18 studies included in the meta-analysis, using subjective PA-related data for tailoring iteration showed a larger effect size than using objective PA data for both subgroups, but neither of the between-group differences were significant (Table 6; *Q_b_*=0.69, P=.40 and *Q_b_*=0, P=.97).

**Table 6:** Subgroup analysis: objective and subjective physical activity measures.

		Subgroup 1: tailored or general^a^				Subgroup 2: tailored or none^a^			
		Studies, n	Sample size, n	Standardized mean difference (95% CI)	P value	Studies, n	Sample size, n	Standardized mean difference (95% CI)	P value
**Test for subgroup differences**									
	Objective	5	1943	0.12 (−0.02 to 0.27)	.10	3	876	0.29 (−0.10 to 0.68)	.14
	Subjective	4	2049	0.20 (0.10 to 0.31)	<.001	7	2260	0.30 (0.10 to 0.50)	.004

^a^Test for subgroup differences: *χ*^2^_1_=0.7, P=.40, *I*^2^=0% for subgroup 1; *χ*^2^_1_=0.0, P=.97, *I*^2^=0%.

In total, 20 out of the 24 studies used iterations for message tailoring. In the studies included in the meta-analysis, only 1 study did not use iteration, so it was not possible to calculate *Q_b_*, only qualitative synthesis on the iteration is presented in this paper. In total, 50% (10/20) of the studies included in the systematic review with iteration showed a positive significant effect size and 25% (1/4) of the studies without iteration showed a positive significant effect size. One study [61] iterated tailored messages based on the determinants of PA (eg, barriers to performing PA and motivational beliefs) obtained from predesigned questions, whereas 19 studies iterated tailored messages based on individuals’ PA-related behavior data (eg, previous steps).

#### Delivery Channels and Message Features

The identified message channels include (1) mobile apps, (2) the web, (3) computer programs, (4) interactive voice response system phone calls, (5) postal printed material, (6) email, and (7) SMS text messages. Nine studies used multiple channels for information dissemination, where postal printed materials and the web were the most used channels among the studies. The forms of message content included text, audio, video, photos, graphs, and hyperlinks, where 14 studies used only text and the remaining 10 studies used multiple forms of messages. For message direction, 1-way messages (unidirectional: participants only receive messages) were applied in 16 studies, and 2-way messages (bidirectional: participants can have interactive communication with senders) were used in the remaining 8 studies. Four different message doses (sending frequency) were found in the studies: high (≥3 times a week), moderate (weekly), low (monthly or longer), and variable (participants decide when to log in for a message). Nine studies used low doses, 4 studies used moderate doses, and 4 studies used high doses. The remaining 7 studies used variable message doses.

### Message Selection Algorithms

The information on message selection algorithms used for generating tailored content provided in the studies was limited. In total, 17 studies did not report information related to computer algorithms, and 5 studies reported that a third-party platform was involved: Overnite (tailored builder) for 4 studies [41,42,58,61] and PropeloTM platform for 1 study [53]. In 6 studies [41,42,44,46,58,61], the stage of change was reported as the decision root (the very first node in a decision tree structure) of the algorithms.

## Discussion

### Principal Findings

This systematic review and meta-analysis aimed to synthesize evidence regarding the effectiveness of CTHC to promote PA for people with or at risk of long-term conditions. Overall, CTHC can be considered as effective, with a small to medium significant effect size for increasing PA among people with or at risk of different long-term conditions. By tailoring the intervention to the individual’s unique needs, preferences, and characteristics, CTHC may be able to address the barriers to, facilitators of, and motivations for PA in a more personalized manner than generic interventions. In contrast to previous suggestions [15], the effect size of CTHC for people with or at risk of long-term conditions was found to be larger than that for tailored messages (including computerized and professional-led tailoring) in promoting PA in the general population [15,16]. However, both the study by Lustria et al [15] (n=6) and our study have a limited number of included papers for individuals with or at risk of long-term conditions. Therefore, further studies on CTHC are required to strengthen our findings. CTHC’s effect size in increasing PA is comparable with that of physiotherapist-led PA interventions for adults with musculoskeletal injury, having or at risk of noncommunicable diseases [68] (standardized mean difference 0.15, 95% CI 0.03-0.27). Thus, CTHC may serve as an effective alternative or complement to traditional physiotherapist-led interventions, particularly for individuals with long-term conditions who face barriers to accessing or adhering to in-person tailored interventions. By tailoring the intervention to the individual’s unique needs and preferences, CTHC may also be able to provide a more personalized and engaging experience that encourages sustained behavior change.

Approximately half of the included studies combined ≥2 behavior change theories and models to construct the tailoring rules. No significant difference was found in the between-groups analysis compared with studies with single or no behavior change theory or models, in contrast to the results obtained by previous studies [10,16].

SCT and stage of change in transtheoretical model are the most commonly used theories and models in the included studies. However, in the studies included in the meta-analysis, no behavioral change theories or models were shown to play a decisive role on treatment effect.

Goal setting and feedback and monitoring were the most commonly used tailoring strategies (22 of 24 studies). Because of the limited information available, it was not possible to determine the effectiveness of these strategies.

For the implementation strategies, no significant difference was found between studies with subjective measurements and studies with objective measurements in either subgroup. Another implementation strategy is iteration, which was used in most of the included studies. In the qualitative analysis, the limited evidence tends to support the use of iteration in CTHC for superior outcomes.

It is important to note that the current analysis was limited by the small number of studies included in the between-groups analysis, which may have reduced the statistical power to detect significant differences. In addition, the specific theories and models used in each study may have varied in their relevance and applicability to the target behavior and population with long-term conditions, which may have influenced the results. Further research is needed to explore the potential benefits and limitations of using different combinations of behavior change theories and models in CTHC interventions to promote PA in individuals with long-term conditions. As more studies become available, it may be possible to identify which specific tailoring components, such as the use of specific BCTs, delivery frequency, and channels, are most effective in promoting PA. This information could help optimize the design and delivery of CTHC interventions and enhance their impact on behavior change in individuals with long-term conditions.

As noted by Mummah et al [69], it is crucial to recognize the key factors for the targeted population when designing CTHC interventions, particularly when targeting individuals with different long-term conditions. Because different behavior change theories emphasize different factors related to behavior change, it is possible that the same theory may have different effects on different populations. However, given the limited number of studies included in this meta-analysis, it is premature to draw conclusions regarding the effective factors for each specific long-term condition. Nonetheless, the factors applied in the studies included in this analysis can serve as a useful reference when designing CTHC interventions to promote PA in individuals with long-term conditions. Future research should continue to explore the underlying mechanisms and moderators of behavior change in CTHC interventions to better tailor these interventions to the needs of specific populations and optimize their effectiveness.

The limited reporting of computer algorithms in the included studies is an important observation as it highlights the need for greater transparency and detail in reporting the technical aspects of CTHC interventions. Only 7 of the studies [41,42,44,46,53,58,61] provided information on the computer algorithms used in their CTHC interventions, and these studies primarily used rule-based systems with stage of change as the decision root for categorizing participants and selecting appropriate messages [44,46]. Although the use of the stage of change as a preferred element for message selection is consistent with the recommendations of Noar et al [70], it is important to note that there may be other factors that could also contribute to the effectiveness of CTHC interventions, such as the use of specific BCTs or the personalization of messages based on individual characteristics. Furthermore, the limited reporting of computer algorithms in the included studies may have also limited the reproducibility and scalability of CTHC interventions in other settings or populations. Without clear and detailed information on the technical aspects of CTHC interventions, it may be difficult for researchers and practitioners to adapt or replicate these interventions in other contexts.

### Strengths and Limitations

The possible limitations of this review should be considered when interpreting the results. First, this review only included studies published in English, which increases the likelihood of omitting relevant research published in other languages. Second, the overall heterogeneity of this meta-analysis was medium to high. Studies included in this review covered different long-term conditions, which may have increased the heterogeneity of the pooled population. Third, the limited number of studies included in the meta-analysis presents a challenge for assessing publication bias. With fewer studies available, it becomes more difficult to identify possible sources of bias and draw reliable conclusions from the analysis. Therefore, caution is needed when interpreting the results, and further research with a larger number of studies is needed to confirm these findings and provide more robust evidence. In addition, nonstandard study designs for behavior change interventions, such as different measures of PA and tailoring strategies, may also increase the heterogeneity of the intervention effects among the included studies. However, as Michie et al [71] suggested, high heterogeneity is common in the meta-analysis of behavior change interventions.

Most of the studies provided only limited information related to behavior change theories and computer algorithms. Specifically, when the studies reported that a combination of behavior change theories was used, details about how theories were combined were often not provided. These absences made it difficult to analyze the effect of tailoring features in detail.

Despite these limitations, all the included studies were RCTs. This review assessed computer-tailored interventions on 1 behavior (PA) among different long-term conditions, which may offer a new perspective for future intervention designers when selecting effective tailoring features.

### Recommendations for Further Study

CTHC studies on increasing PA in people with or at risk of long-term conditions are still very limited. It is still difficult to make suggestions related to CTHC design for future studies. On the basis of the limited information obtained from this review, the stage of change could be considered as the root of the decision rules for participant and message categorization. An individual’s stage of change in increasing PA can be used to match the tailored messages for the same stage initially. Within each stage, different determinants of performing PA can be promoted by emphasizing different types of information (eg, benefits of increasing PA for individuals in stage 1; behavior data compared with goals for individuals in stages 3 and 4).

Iteration is also suggested as an advantageous implementation strategy. With iteration, future message content can be adjusted based on individuals’ responses to previous messages. In addition to the participants’ PA performance data, iterations can be combined with other tailoring strategies, such as the participants’ preferences regarding the messages’ content, participants’ preferences regarding the message sources, participants’ objective status, and inner values. Iterating only on behavior performance might still lead to a low use rate or high attrition rate if the message content is not sufficiently attractive to the recipients. Future studies could include recommendation systems supported by machine learning to match tailored messages to participants’ preferences regarding the message itself. These new approaches have drawn more attention in recent behavior change studies [68-72].

Most studies have assessed short-term effectiveness. There were 7 studies that assessed outcomes over 12 months, and most of them obtained nonsignificant results. Future studies should assess the results over a longer period to understand the long-term effects of tailored messages.

The lack of sufficient information related to the CTHC design was a major challenge in evaluating the effectiveness of the tailoring elements in the included studies. More detailed reporting of behavior change theories and models, BCTs, and computer algorithms for message selection in future CTHC studies would be invaluable for understanding how CTHC works in behavior change interventions.

This analysis underscores the importance of transparent and detailed reporting of the technical aspects of CTHC interventions, particularly the computer algorithms used for message selection. Providing clear information on the decision rules, message selection criteria, and personalization methods can improve the reproducibility, scalability, and effectiveness of CTHC interventions for promoting PA in individuals with long-term conditions.

Therefore, it is critical for future studies to prioritize the transparent reporting of CTHC design elements, as this can enable researchers and practitioners in diverse settings to adapt or replicate these interventions with greater confidence. By improving the clarity and detail of reporting, we can enhance our understanding of the underlying mechanisms and moderators of behavior change in CTHC interventions and ultimately optimize their impact on PA behaviors in individuals with long-term conditions.

### Conclusions

Overall, the CTHC intervention was shown to be effective in increasing PA levels in people with or at risk of different long-term conditions. Most CTHC studies aimed to provide tailored PA goal setting, feedback, and suggestions based on individuals’ PA-related data. With the support of behavior change theories and models, CTHC studies also targeted individuals’ intentions, motivations, barriers, and preferences. Although no behavior change theory or model was found to be superior to others, using the stage of change as the root of decision rules for message selection could be beneficial. In terms of the message implementation strategy, iteration appears to be an effective element of CTHC. Owing to the limited information available related to tailored content design and computer algorithms, it is still challenging to synthesize or conclude which tailoring elements are effective and why. More CTHC studies are needed for a comprehensive evaluation in the future.

## Appendix

Multimedia Appendix 1 Search strategy.

Multimedia Appendix 2 PRISMA (Preferred Reporting Items for Systematic Reviews and Meta-Analyses) checklist.

Multimedia Appendix 3 Publication bias analysis.

## Abbreviations

BCT: behavior change technique
CTHC: computer-tailored health communication
HAPA: health action process approach
PA: physical activity
PRISMA: Preferred Reporting Items for Systematic Reviews and Meta-Analyses
RCT: randomized controlled trial
SCT: social cognitive theory

## Data Availability

The data sets generated and analyzed during this study are available from the corresponding author upon reasonable request. However, all data were generated based on the original data from the included studies, making it easy to extract data accordingly.
