# Supplementary material for: Effectiveness of Computer-Tailored Health Communication in Increasing Physical Activity in People With or at Risk of Long-Term Conditions: Systematic Review and Meta-Analysis
Source: J Med Internet Res. 2023 Oct 4;25:e46622. doi: 10.2196/46622 (PMC10585448; doi:10.2196/46622)
Supplement: Multimedia Appendix 1 [file jmir_v25i1e46622_app1.docx]

| 1. | exp Exercise/ or exp Exercise Therapy/ |
| --- | --- |
| 2. | exp "Physical and Rehabilitation Medicine"/ |
| 3. | physical activit*.tw. |
| 4. | 1 or 2 or 3 |
| 5. | exp Chronic Disease/ |
| 6. | exp Diabetes Mellitus, Type 2/ |
| 7. | type 2 diabetes.tw. |
| 8. | T2 diabetes.tw. |
| 9. | type II diabetes.tw. |
| 10. | 6 or 7 or 8 or 9 |
| 11. | exp Osteoarthritis/ or exp Osteoarthritis, Knee/ or exp Arthritis/ or exp Arthritis, Experimental/ or exp Arthritis, Rheumatoid/ |
| 12. | exp Asthma/ or exp Asthma-Chronic Obstructive Pulmonary Disease Overlap Syndrome/ |
| 13. | exp Neoplasms/ |
| 14. | exp Cardiovascular Diseases/ |
| 15. | Renal Insufficiency, Chronic/ |
| 16. | exp Pulmonary Disease, Chronic Obstructive/ |
| 17. | COPD.tw. |
| 18. | 16 or 17 |
| 19. | exp Dyslipidemias/ |
| 20. | exp Chronic Pain/ or exp Fibromyalgia/ |
| 21. | exp Frailty/ |
| 22. | exp Heart Diseases/ |
| 23. | exp Hypertension/ |
| 24. | exp Obesity/ |
| 25. | 5 or 10 or 11 or 12 or 13 or 14 or 15 or 18 or 19 or 20 or 21 or 22 or 23 or 24 |
| 26. | exp Health Information Systems/ |
| 27. | exp Health Education/ or exp Health Promotion/ or exp Health Knowledge, Attitudes, Practice/ or exp Internet/ |
| 28. | exp Health Knowledge, Attitudes, Practice/ or exp Patient Education as Topic/ |
| 29. | exp Health Promotion/ or exp Text Messaging/ |
| 30. | exp Cognitive Behavioral Therapy/ |
| 31. | exp Information Dissemination/ |
| 32. | 26 or 27 or 28 or 29 or 30 or 31 |
| 33. | exp Randomized Controlled Trials as Topic/ |
| 34. | randomized controlled trial.pt. |
| 35. | clinical trial.pt. |
| 36. | controlled clinical trial.pt. |
| 37. | 33 or 34 or 35 or 36 |
| 38. | (tailored adj2 information).tw. |
| 39. | (tailoring adj2 information).tw. |
| 40. | (tailored adj2 feedback*).tw. |
| 41. | (tailoring adj2 feedback*).tw. |
| 42. | (tailored adj2 messag*).tw. |
| 43. | (tailoring adj2 messag*).tw. |
| 44. | (tailor* adj2 (communication or advice*)).tw. |
| 45. | (tailor* adj2 material*).tw. |
| 46. | (tailor* adj2 intervention*).tw. |
| 47. | (computer adj2 tailor*).tw. |
| 48. | (personali* adj2 (information or communication or feedback or message* or advice* or material*)).tw. |
| 49. | 38 or 39 or 40 or 41 or 42 or 43 or 44 or 45 or 46 or 47 |
| 50. | 48 or 49 |
| 51. | 25 and 37 and 50 |
| 52. | tailor*.tw. |
| 53. | 4 and 25 and 37 and 50 |
| 54. | 32 and 52 |
| 55. | 50 or 54 |
| 56. | 25 and 37 and 55 |
| 57. | 4 and 56 |
