# Supplementary material for: Effectiveness of Computer-Tailored Health Communication in Increasing Physical Activity in People With or at Risk of Long-Term Conditions: Systematic Review and Meta-Analysis
Source: J Med Internet Res. 2023 Oct 4;25:e46622. doi: 10.2196/46622 (PMC10585448; doi:10.2196/46622)

meta bias, egger random(reml)

Effect-size label: Effect size

Effect size: smd

Std. err.: _meta_se

Regression-based Egger test for small-study effects

Random-effects model

Method: REML

H0: beta1 = 0; no small-study effects

beta1 = 2.75

SE of beta1 = 1.163

z = 2.36

Prob > |z| = 0.0182

. meta trimfill

Effect-size label: Effect size

Effect size: smd

Std. err.: _meta_se

Nonparametric trim-and-fill analysis of publication bias

Linear estimator, imputing on the left

Iteration Number of studies = 10

Model: Random-effects observed = 9

Method: REML imputed = 1

Pooling

Model: Random-effects

Method: REML

---------------------------------------------------------------

Studies | Effect size [95% conf. interval]

---------------------+-----------------------------------------

Observed | 0.159 0.068 0.250

Observed + Imputed | 0.139 0.036 0.242

---------------------------------------------------------------

.

Funnel plot (Tailored messages VS General messages)


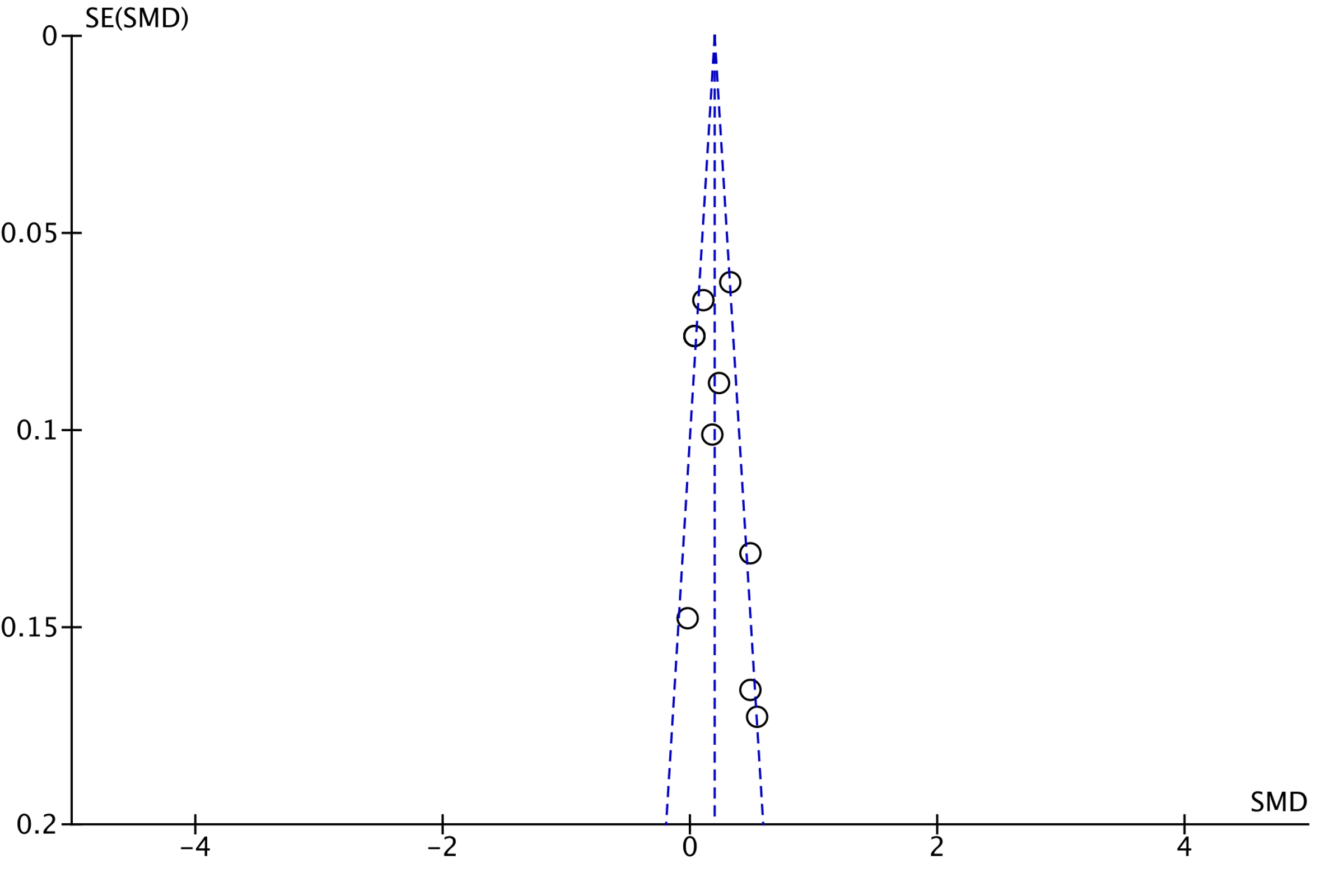


Funnel plot (Tailored messages VS None messages)


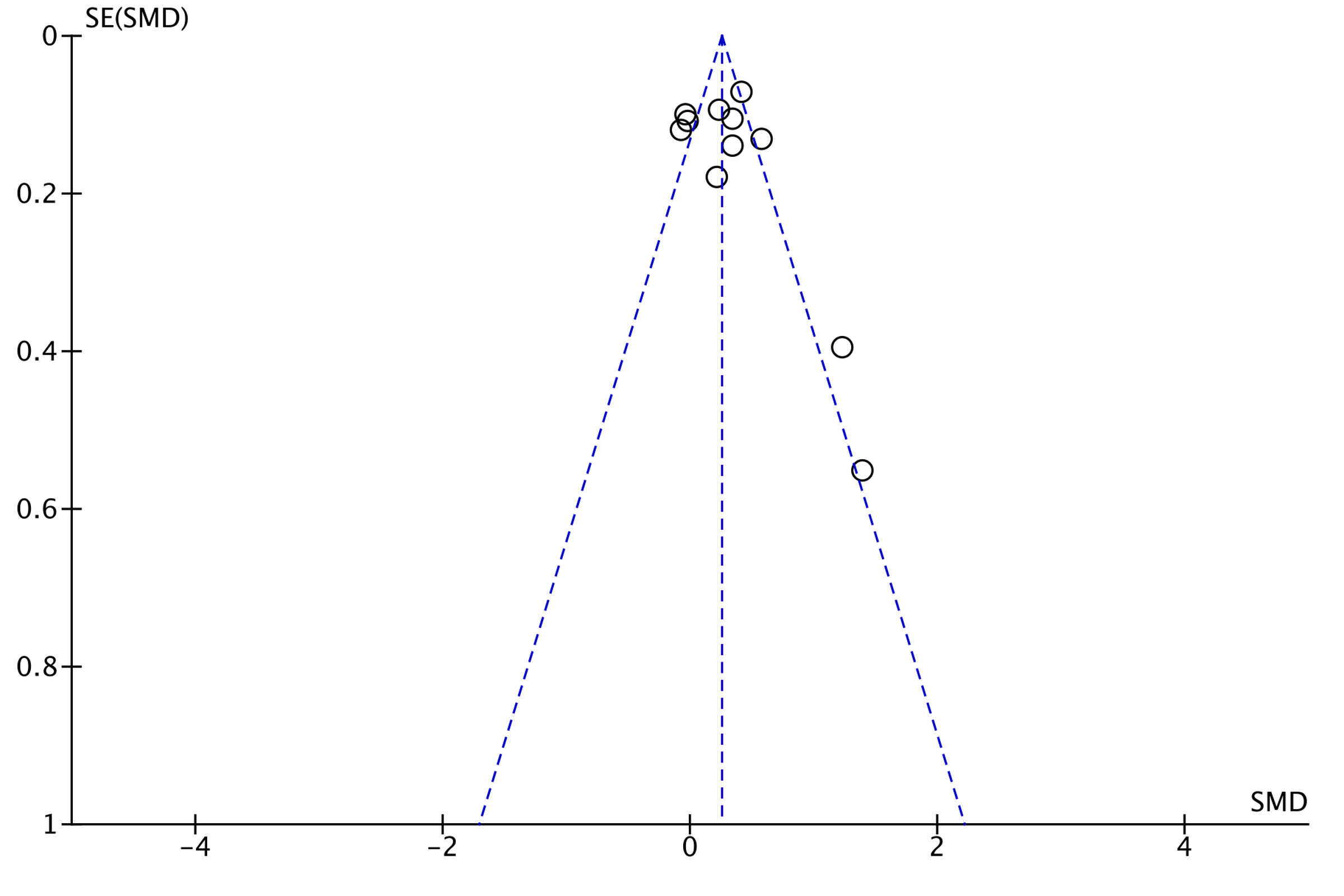

Supplement: Multimedia Appendix 3 [file jmir_v25i1e46622_app3.docx]
